# Overexpression of *AmRosea1* Gene Confers Drought and Salt Tolerance in Rice

**DOI:** 10.3390/ijms18010002

**Published:** 2016-12-22

**Authors:** Mingzhu Dou, Sanhong Fan, Suxin Yang, Rongfeng Huang, Huiyun Yu, Xianzhong Feng

**Affiliations:** 1 Key Laboratory of Plant Stress Research, College of Life Sciences, Shandong Normal University, Jinan 250014, China; kadmz@163.com; 2 Key Laboratory of Soybean Molecular Design Breeding, Northeast Institute of Geography and Agroecology, Chinese Academy of Sciences, Changchun 130102, China; yangsuxin@iga.ac.cn; 3 State Key Laboratory of Crop Stress Biology for Arid Areas, College of Life Sciences, Northwest A&F University, Yangling 712100, China; shfan@nwsuaf.edu.cn (S.F.); 18829782586@163.com (H.Y.); 4 Shanghai Center for Plant Stress Biology, Shanghai Institutes for Biological Sciences, Chinese Academy of Sciences, Shanghai 201602, China; huang_rfeng@126.com

**Keywords:** *AmRosea1* gene, transcriptome analysis, drought tolerance, salt tolerance, rice

## Abstract

Ectopic expression of the MYB transcription factor of AmROSEA1 from *Antirrhinum majus* has been reported to change anthocyanin and other metabolites in several species. In this study, we found that overexpression of *AmRosea1* significantly improved the tolerance of transgenic rice to drought and salinity stresses. Transcriptome analysis revealed that a considerable number of stress-related genes were affected by exogenous *AmRosea1* during both drought and salinity stress treatments. These affected genes are involved in stress signal transduction, the hormone signal pathway, ion homeostasis and the enzymes that remove peroxides. This work suggests that the *AmRosea1* gene is a potential candidate for genetic engineering of crops.

## 1. Introduction

Anthocyanins are pigments that belong to the flavonoid class and are synthesized via the phenylpropanoid pathway, which are responsible for many colors, such as red, purple, and blue in leaves, flowers, and fruits of angiosperms. The activity of R2R3-MYB genes is probably the major cause of natural variation in anthocyanin production in various plants [1,2,3,4,5,6,7,8,9,10,11,12]. The variation of anthocyanin pattern and intensity between more than six *Antirrhinum majus* species are attributable to differences of the activity in the *Rosea* and *Venosa* loci, which encode R2R3-MYB transcription factors. The *Rosea* locus, containing two adjutant genes, *AmRosea1* and *AmRosea2*, regulates the spatial distribution and intensity of pigmentation in both the corolla tubes and lobes of *A. majus*; and the *Venosa* locus is related to the pigmentation pattern of venation in the corolla. The expression of flavanone 3-hydroxylase (*F3H*), flavonol synthase (*FLS*), flavonoid 3’-hydroxylase (*F3ʹH*), dihydroflavonol 4-reductase (*DFR*), leucoanthocyanidin dioxygenase (*LDOX*), and UDP-glucose 3-O-flavonoid transferase (*UFGT*) genes are highly dependent on AmROSEA1. On the other hand, AmROSEA2 controls only the *CHI* and *F3’H* genes [11,13].

R2R3-MYB proteins are not only involved in the primary and secondary metabolism, but are also involved in developmental processes and the responses to biotic and abiotic stresses. AtMYB2 protein up-regulated the expression of ABA-inducible genes in drought-treated *Arabidopsis* plants [14], and overexpression of AtMYB15 enhanced the drought tolerance of transgenic *Arabidopsis* [15]. Rice OsMYB2 and OsMYB4 proteins had been shown to have functions in cold and dehydration tolerance [16,17]. Several wheat R2R3-MYBs, including TaMYB1 [18], TaMYB2A [19], TaMYB33 [20], TaMYB73 [21], and TaMYB30-B [22], were shown to improve stress tolerance in transgenic plants.

Ectopic expression of transcription factors AmROSEA1 and AmDELILA from *Antirrhinum majus* under fruit-specific E8 promoter in tomato resulted in substantial accumulation of anthocyanins throughout the fruit [23]. When *AmRosea1* and *AmDELILA* were constitutively expressed under the 35S promoter in *Nicotiana benthamiana*, a single anthocyanin, delphinidin-3-rutinoside (D3R), was robustly induced. Surprisingly, except for the D3R, a range of additional metabolites which were not synthesized in the flavonoid pathway were also strongly up-regulated. Most notable among these additional metabolites were nornicotine conjugates with butanoyl, hexanoyl, and octanoyl hydrophobic moieties and phenolamides. The study showed that the effect of ROS1 and DEL expression in *N. benthamiana* leaves extended beyond the flavonoid pathway [24].

AmDELILA is not necessary for AmROSEA1 function in some species. AmROSEA1 is essential for inducing accumulation of different compounds in *N. benthamiana*, while AmDELILA is only required to potentiate the functions of AmROSEA1 [24]. Similarly, the expression of AmROSEA1 alone could mediate anthocyanin formation in tomatoes, but AmDELILA was not necessary [25]. When *35S:AmRosea1* was introduced to lisianthus, temporal, spatial and quantitative changes in anthocyanin pigmentation were observed. In the stems of *35S:AmRosea1 Petunia* line MP [Petunia axillaris × (P. axillaris × P. hybrida)], anthocyanin pigmentation was enhanced [26]. The mature green leaves of green leaf cotton variety CCRI 24 (*Gossypium hirsutum* L.) accumulated red anthocyanin after being infiltrated by *Agrobacterium* strain GV3101/pBI35S::*AMRosea1* [27].

Although *AmRosea1* has been introduced into several dicotyledonous species, there have been no studies reported on the ectopic expression of *AmRosea1* in rice (*Oryza sativa* L.), which is an important model plant of monocotyledons and grain crop that provides food for more than half of the human population. In this study, we introduced *AmRosea1* into rice, and examined the anthocyanin content and stress tolerance of transgenic plants. We found that AmROSEA1 could not effectively induce anthocyanin biosynthesis in rice, but clearly improved the tolerance of transgenic rice to drought and salinity stresses. We used Illumina sequencing technology to analyze the transcriptomes of wild type (WT) and transgenic rice plants under drought and salinity conditions and discovered that the transcriptional level of a considerable number of stress-related genes was affected by exogenous *AmRosea1*. These affected genes were involved in stress signal transduction, the hormone signal pathway, ion balance, and the removal of reactive oxygen species (ROS).

## 2. Results

### 2.1. Generation of Transgenic Rice Plants by Overexpressing AmRosea1

**Figure 1:**
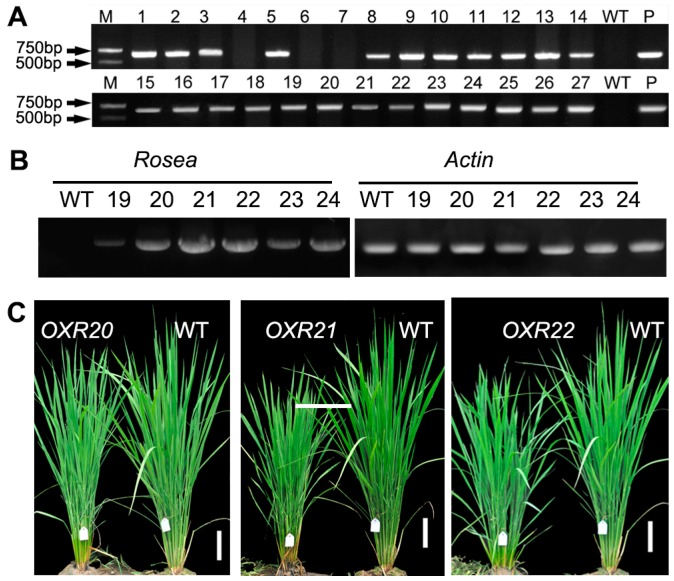
Expression analysis of *AmRosea1* in transgenic rice plants and plant height. (**A**) PCR analysis of transgenic plants: positive transgenic lines and positive control have a 675-bp PCR band; M: molecular marker; P: pCAMBIA1300-*AmRosea1* plasmid DNA as template; (**B**) *AmRosea1* transcript levels in the transgenic lines, as revealed by semi-quantitative real-time (RT)-PCR analysis. *Osactin1* was used as the internal control (left); (**C**) overall morphology of three-month-old wild-type and homozygous 35S:*AmRosea1* transgenic rice plants (independent lines: OXR20, OXR21, and OXR22). OXR plants display a growth retardation phenotype. Scale bar: 10 cm. WT: wild-type plant, OXR: *AmRosea1* over-expression plant.

We cloned the snapdragon gene *AmRosea1*, containing a 660-bp open reading frame (ORF) and encoding a 220-amino acid MYB-related transcription factor, from flowers of snapdragon *A. majus* into the pBluescriptII KS vector. In order to generate transgenic rice plants that carried the *AmRosea1* gene, we constructed a binary vector pCAMBIA1300-*AmRosea1*, where *AmRosea1* cDNA was cloned under the 35S promoter (Figure S1). Twenty-seven independent event lines, labeled as *AmRosea1* over-expression plant (OXR) 1-27, were produced through *Agrobacterium-*mediated rice transformation. PCR analysis using *AmRosea1*-specific primers confirmed the presence of the transgene in 24 T0 lines (Figure 1A). The positive transgenic lines and positive control have 675 bp bands, but the WT line and negative control were not found to have any band.

We investigated the expression of *AmRosea1* in the T0-generation transgenic lines by semi-quantitative RT-PCR. The results showed that WT had no expression of *AmRosea1*, and OXR20, OXR21, and OXR22 lines exhibited higher expression of *AmRosea1* than OXR19, OXR23, and OXR24 (Figure 1B). Herbicide resistance of the progeny of line OXR20, OXR21, and OXR22 were tested in hygromycin-containing medium. One-hundred seeds of each line were screened, and 61 progenies of OXR20, 68 of OXR21, and 64 of OXR22 survived. These results indicated that these three lines were all inserted by a single copy of *AmRosea1* gene. We finally chose their homozygote progenies for a subsequent experiment. There was no obvious phenotypic difference between transgenic seedlings and WT rice seedlings except that the mature transgenic rice plants were clearly shorter than the WT plants (Figure 1C).

To test whether ectopic expression of *AmRosea1* in rice affects anthocyanin accumulation, we analyzed the content of anthocyanin in mature seeds and leaves at two developmental stages of rice, trefoil and heading. No anthocyanin accumulation was detected in either young leaves or mature seeds of WT and the tested transgenic lines, and no clear differences of anthocyanin accumulation were observed in their mature leaves (Figure S2). In mature seeds, young leaves, and mature leaves, there was no significant difference of flavonoid content between the WT and the transgenic lines (Figure S2). These results indicated that ectopic expression of *AmRosea1* did not change the content of anthocyanin and flavonoid in tested transgenic plants.

### 2.2. The Relationship between Overexpression of AmRosea1 and Improvement of Tolerance to Drought and Salt Stresses in Transgenic Rice

**Figure 2:**
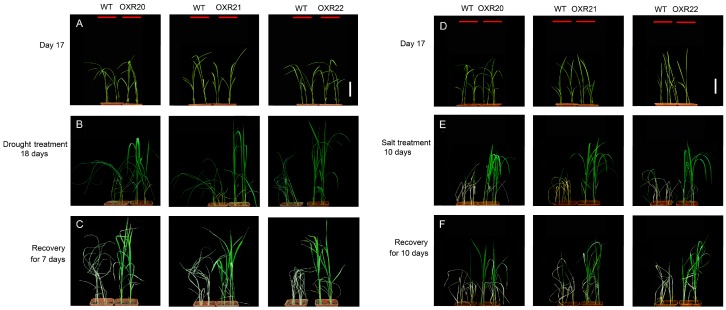
Phenotypes of WT and OXR rice plants with drought and salt treatments in greenhouse. (**A**) 17 days after germination before drought stress treatment; (**B**) 35 days after germination and drought stress treatment for 18 days; (**C**) recovery of drought stress treatment for 7 days; (**D**) 17 days after germination before salt stress treatment; (**E**) 27 days after germination and salt stress treatment for 10 days; (**F**) recovery of salt stress treatment for 10 days. Scale bar = 5 cm.

To examine the function of *AmRosea1* in transgenic rice, drought and salt stresses experiments were carried out. Before the drought stress treatment, both the WT and transgenic plants grew normally (Figure 2A). WT plants began to wither, but the tested transgenic plants grew vigorously after 14 days without watering. WT plants showed severe leaf wilting or rolling after 18 days without watering, while the transgenic lines remained vigorous (Figure 2B). After rewatering for seven days, a large difference in the survival ratio of WT and transgenic plants was seen: the survival rates of three tested transgenic lines, OXR20, OXR21, and OXR22, were 78%, 85%, and 80% respectively (*n* = 24); all of which were higher than the 20.8% survival rate of WT plants (Figure 2C). These results showed that AmROSEA1 enhanced tolerance to drought stress in transgenic rice lines.

After being treated with NaCl solutions for 10 days, all WT plants were severely withered and turned white. By contrast, the transgenic lines were still vigorous although the leaf tip did wither (Figure 2E). After recovering in normal tap water for 10 days, all WT plants died, whereas more than 70% of OXR20, 75% of OXR21, and 69% of OXR22 seedlings remained vigorous (*n* = 24) (Figure 2F). This data indicated that AmROSEA1 enhanced tolerance to salt stress in transgenic rice lines. Taken together, the above results demonstrated that overexpression of *AmRosea1* conferred transgenic rice lines significant tolerance to both drought and salt stresses.

### 2.3. Quantification of Transcripts and Identification of Differentially Expressed Genes through Transcriptome Analysis

In order to investigate the molecular mechanism underlying the salt-tolerance and drought-tolerance of transgenic lines, transgenic line OXR21 was chosen for RNA-seq analysis as the representative of the ectopic expression lines. The number of clean reads per library ranged from 7.12 to 7.40 million (Table S1). The proportion of unambiguously mapped reads per library ranged from 84.15% (OXR_S1) to 91.58% (WT_S6), and the proportion of unique matches ranged from 42.89% (WT_P24) to 49.47% (WT_S6).

**Figure 3:**
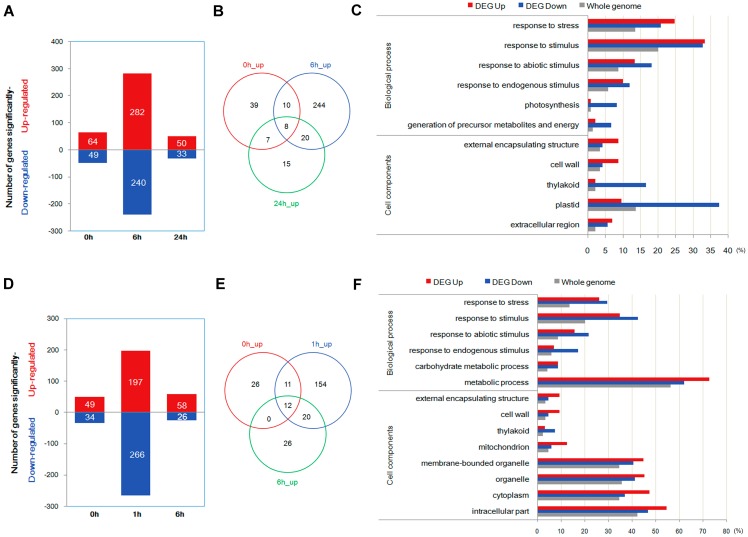
(**A**) Number of differently expressed genes (DEGs) in 35S:*AmRosea1* transgenic plants line OXR21 relative to the expression level of wild-type plants (WT) at each point in time (0, 6, and 24 h) of polyethylene glycol (PEG) treatment (*p* < 0.05, corrected *p*-value of false discovery rate, FDR < 0.05); (**B**) a Venn diagram analysis of up-regulated genes in 35S*:AmRosea1* transgenic plants line OXR21 compared with WT rice plants for different durations of PEG treatment (0, 6, 24 h); (**C**) GO (Gene Ontology) classification of DEGs of salt group using AgriGO; (**D**) number of differently expressed genes in 35S*:AmRosea1* transgenic plants line OXR21 relative to the expression level of wild-type plants (WT) at each time point (0, 6, and 24 h) of salt treatment (*p* < 0.05, corrected *p*-value of FDR < 0.05); (**E**) a Venn diagram analysis of up-regulated genes in 35S*:AmRosea1* transgenic plants line compared with WT rice plants for different durations of salt treatment (0, 1, and 6 h); (**F**) GO classification of DEGs of salt group using AgriGO. Only GO terms of DEGs with significance (*p* < 0.05, corrected *p*-value of FDR < 0.05) are presented when comparing those of the rice whole genome.

Analysis of differentially expressed genes (DEGs) showed that 64, 282, and 50 genes were up-regulated, and 49, 240, and 33 genes were down-regulated in the transgenic line compared to the WT plants at 0, 6, and 24 h of polyethylene glycol (PEG) treatment, respectively (Figure 3A). In addition, 49, 197, and 58 genes were up-regulated, and 34, 266, and 26 genes were down-regulated in the transgenic line compared to the WT plants at 0, 1, and 6 h of salt treatment, respectively (Figure 3D). The details on DEGs are shown in Tables S2 and S3.

Venn diagram (Figure 3B) analysis showed three types of genes related to drought stress: (1) Eight genes were up-regulated at all three PEG treatment-tested points in time, which might have been consistently caused by transgenic events; (2) Twenty were up-regulated both at 6 h and 24 h of PEG treatment, which were more likely to contribute to drought tolerance; (3) Fifteen were up-regulated at 24 h of PEG treatment, which might maintain the drought-tolerance characteristics in transgenic plants. As shown in supplementary Table S4, peroxidase, RD22, and submergence-induced protein were included in the eight genes of the first category; peroxidase, lipoxygenase, myo-inositol oxygenase, glycine-rich cell wall structural protein precursor, WRKY transcription factor 62, water-stress inducible protein RAB16A, ethylene response factor, and late embryogenesis abundant (LEA)-like protein were included in the 20 genes of the second category; and fatty acid α-dioxygenase, high-affinity potassium transporter, MYB21, and germin-like protein 8–11 (disease resistance) were included in the 15 genes of the last category.

Venn diagram analysis (Figure 3E and Table S4) also showed three types of genes related to salt stress: (1) Twelve transgenic event related genes were up-regulated at all three salt treatment-tested points in time, including MYB-related transcription factor, CBL (Calcineurin B-like protein) -interacting protein kinase, protein disulfide isomerase, and submergence-induced protein; (2) Twenty promoting salt tolerance genes were up-regulated both at 1 h and 6 h of salt treatment, including cellulose synthase, potassium transporter, heat shock protein, and glycine-rich cell wall structural protein; (3) Twenty-six salt tolerance related genes were up-regulated at 6 h of salt treatment, comprising glutathione S-transferase (GST), peroxidase, trehalose-6-phosphate synthase, cellulose synthase-like H1, heat shock protein (HSP), serine/threonine protein kinase, and catalase isozyme B.

Gene Ontology (GO) analysis on the DEGs of the drought-stress group and the salt-stress group revealed that 68.03% and 71.1% of these DEGs were annotated with various GO biological terms, respectively (Figure 3C,F). Within the category “biological processes”, six GO terms were enriched in the drought stress DEGs and salt stress DEGs, respectively. Four GO terms enriched in the sets of DEGs from the PEG group were also enriched in the salt group; they are “response to endogenous stimulus”, “response to abiotic stimulus”, “response to stress”, and “response to stimulus”, which were all related to stimulus. Meanwhile, for the cell components category, three GO terms enriched in DEGs for the drought-stress group were also enriched in DEGs for the salt-stress group. The same GO terms of cell components are “thylakoid”, “cell wall”, and “external encapsulating structure”. The above results showed that the same mechanism might be employed in both salt tolerance and drought tolerance of the 35S*:AmRosea1* rice plants. In addition to the same above GO terms, “photosynthesis”, “generation of precursor metabolites and energy”, “plastid”, and “extracellular region” were also enriched in DEGs for the PEG-stress group; “carbohydrate metabolic process”, “metabolic process” “mitochondrion”, “membrane-bounded organelle” “organelle” “cytoplasm” and “intracellular part” were also enriched in DEGs for the salt-stress group (Figure 3C,F).

### 2.4. DEGs Related to Hormone Signal Transduction Pathways

Among the 610 differently transcribed genes in transgenic plants compared to WT plants under drought conditions, 109 genes were assigned to KEGG (Kyoto Encyclopedia of Genes and Genomes) orthologs, with eight DEGs mapping to hormone signal transduction pathway (Table S5). Next, 126 of the 557 DEGs under salinity stress were assigned to KEGG orthologs, with nine DEGs mapping to hormone signal transduction pathways (Table S6). These genes encoding proteins of cytokinine, abscisic acid, ethylene, jasmonic acid, and salicylic acid pathways expressed differently in transgenic plants compared to WT plants under drought or salt stress. The fold change of DEGs involved in hormone signal pathways are listed in Tables S5 and S6.

**Figure 4:**
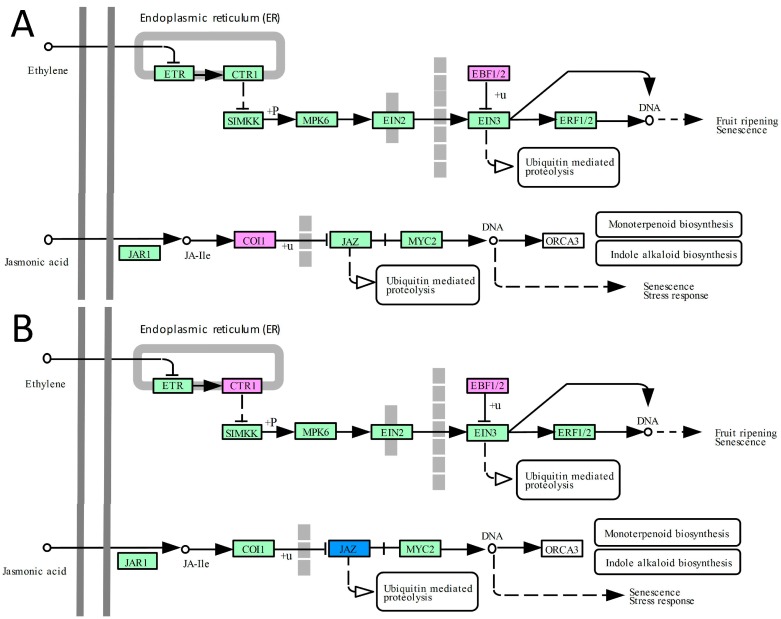
KEGG pathway analysis of ethylene signal and JA signal pathways. Each rectangular box represents genes that are mapped to *Oryza sativa* in the species-specific pathway map. Boxes with pink background indicate the corresponding genes were up-regulated in the OXR samples compared with WT samples at some tested point in time, as determined by RNA-seq. Blue background indicates down-regulated and green background indicates corresponding genes did not exhibit obvious expression differences at any tested time point between OXR samples and WT samples. (**A**) PEG treatment group; (**B**) salt treatment group. The arrows with white colors indicate linking to another map; arrows with black color indicate molecular interaction or relation.

KEGG pathway analysis results of both ethylene signal and JA signal pathways are shown in Figure 4. One gene (Os06t0605900-01), encoding EBF1, was up-regulated under both drought and salt stresses; another gene (Os02t0527600-01), encoding CTR1, was up-regulated under salt stress in transgenic plants compared to WT plants. These differences of gene expression repressed the ethylene signal transduction pathway in transgenic rice plants (Figure 4). Under drought stress, one gene (Os05t0449500-01) encoding COI1 was up-regulated and under salt stress, and two genes (Os03t0402800-01 and Os07t0615200-01) encoding JAZ were down-regulated in transgenic plants compared to WT plants. In general, the JAZ signal transduction pathway was promoted in transgenic plants compared to WT plants under both drought and salt stresses (Figure 4).

### 2.5. DEGs Related to Protection from Osmotic Damage

High salt concentration and water deficit lead to hyperosmotic stress, which destroys membrane and protein structures. Adjusting ion transport and accumulating osmolytes are effective mechanisms for plants to defend against hyperosmotic stress and maintain normal metabolic activities. Compared with WT plants, Os09t0448200-02, similar to high-affinity potassium transporter, was up-regulated under drought stress; and Os07t0669700-01 (encoding potassium transporter 4), was up-regulated under salt stress. With the exception of Os09t0376800-01 (*OsTPS11*), most DEGs related to accumulating osmolytes, such as fructosyltransferase (Os02t0106100-01, Os03t0316200-01, Os01t0170000-01), are down-regulated (Table 1 and Table 2). These results suggested that the tolerance of transgenic plants was more likely from regulation of ion balance other than accumulation of osmolytes.

### 2.6. DEGs Related to Protection from Oxidative Damage

Reactive oxygen species (ROS) induced by stress can destroy cell membranes, proteins, and nucleic acids [28,29]. The accumulation of ROS such as superoxide and hydroxyl radicals is largely counteracted by an intricate antioxidant defense system [30,31]. Three catalases (CATs) and fifteen peroxidases (PODs) are mainly up-regulated in transgenic plants compared to WT under drought and salt treatments (Table 3 and Table 4). However, one ascorbate peroxidase (APX; Os04t0602100-01) and one GST (Os04t0435500-01) were always down-regulated in transgenic plants with both stresses. These results suggested that protection from oxidative damage might be the main reason for causing stress tolerance in transgenic plants.

In addition to the above DEGs, some previously reported stress-relating genes were also identified. Under drought stress, 6 heat shock proteins (4 up, 2 down), 2 late embryogenesis abundant proteins (LEA; 1 up, 1 down), 2 chaperone proteins (2 up, all) and 2 metallothionein proteins (2 down, all) were expressed differently between WT and transgenic plants (Table S7). Under high salt conditions, 7 heat shock proteins (HSP; 6 up, 1 down), 5 LEA proteins (1 up, 4 down), 1 chaperone protein (down), 1 metallothionein protein1 (up) and 1 plasma membrane intrinsic protein (PIP) were also expressed differently between WT and transgenic plants (Table S8). One HSP (Os01t0135900-02) and one LEA protein (Os01t0314800-01) showed similar regulated expression patterns under both drought and salt stresses; this indicated that some proteins might be shared in both stresses.

### 2.7. Verification of Differential Transcription Using Quantitative Real Time PCR (qRT-PCR)

**Figure 5:**
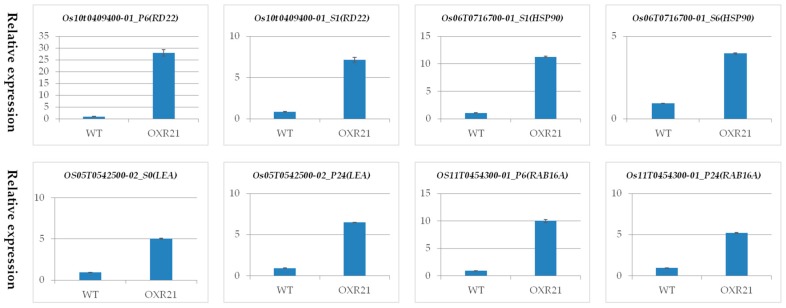

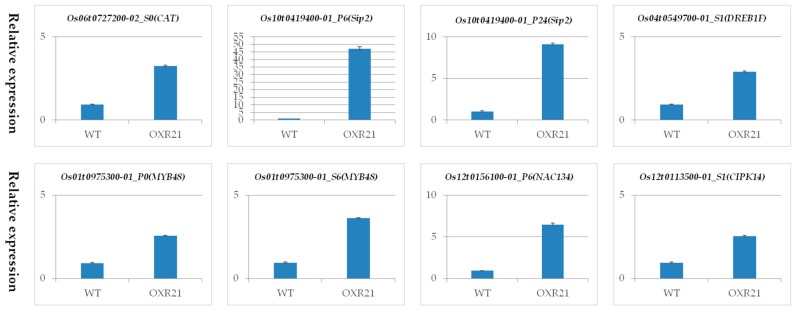
Quantitative PCR (qPCR) validation for DEGs identified by RNA-seq in the comparison between OXR21 and WT. S0, S1 and S6 represent salt treatment for 0, 1, and 6 h, respectively. P0, P6 and P24 represent PEG treatment for 0, 6, and 24 h, respectively.

To validate the expression profiles of genes in RNA-seq results, we performed qRT-PCR on a few DEGs, including stress responsible genes, *RD22* (Os10t0409400-01), heat shock protein (*HSP90*, Os06t0716700-01), LEA protein (Os05t0542500-02), *RAB16A* (Os11t0454300-01), catalase (*CAT*, Os06t0727200-02) and submergence-induced protein 2 (*Sip2*, Os10t0419400-01); transcriptional factors, dehydration responsive element binding protein 1F (*DREB1F*, Os04t0549700-01), *MYB48* (Os01t0975300-01) and *NAC134* (Os12t0156100-01); and serine-threonine kinase, CBL-interacting protein kinase 14 (*CIPK14*, Os12t0113500-01). The qPCR outcomes correlated closely with the transcript abundances estimated from the RNA-seq output, and all the confirmed qPCR results in Figure 5 supported the defense mechanisms against drought and salinity in transgenic plants.

## 3. Discussion

### 3.1. The Abiotic Tolerance of Transgenic Plants Extends AmROSEA1 Function beyond the Flavonoid Pathway

The fact that R2R3-MYB factors regulate multiple physiological processes in plants and that they mostly act as heterodimers with bHLH (basic helix-loop-helix) transcription factors allows for a high degree of plasticity in the control they exert and in the effect of ectopic expression. Although AmROSEA1 was not required for the accumulation of transcripts of the gene *CHS* in *Antirrhinum*, it elevated *CHS* transcript levels in petunia and lisianthus [11,13]. The expression of AmROSEA1 in *N. benthamiana* leaves up-regulated nornicotine conjugates, which extend beyond the flavonoid pathway [24]. There is no visual difference in anthocyanin accumulation between transgenic plants and WT plants. The transcriptome result demonstrated that overexpression of *AmRosea1* alone was unable to markedly transcriptionally up-regulate *F3H*, *F3’H*, *FLS*, *DFR*, and *UFGT*, which were the target genes of AmROSEA1 in snapdragon *A. majus*, and other flavonoid biosynthetic genes in leaves (Tables S9 and S10). This implies that the anthocyanin regulatory mechanisms in monocotyledonous rice may differ from those of dicotyledonous snapdragon.

### 3.2. Some Unknown Function Genes and Several Abiotic Stress Response Genes Might Directly Relate to Abiotic Tolerance of Transgenic Plants

Through transcriptome analysis, we found 343 up-regulated and 278 down-regulated *DEGs* under drought treatment, and 249 up-regulated and 312 down-regulated DEGs under salt treatment (Figure 3). Three common up-regulated genes at all tested points in time of both drought and salt treatments are Os07t0125500-01, Os07t0126301-01 and Os10t0419400-01 (Table S4). Os07t0125500-01 and Os07t0126301-01 belong to allergen V5/Tpx-1 related family protein. Os10t0419400-01 is similar to submergence-induced protein 2. Although the functions of these three genes have not been reported yet, from current bioinformatics analysis they all seem to be related to the stress response. The further investigation of above unknown gene functions and the relationship of other factors will provide evidence to interpret the reason for the abiotic tolerance of transgenic plants, which might also give some hints on how the plant deals with the integration of foreign ectopic genes.

With the exception of the above genes, abiotic stress response genes were also revealed to be up-regulated under either drought or salt treatment, such as Os01t0975300-01 (*OsMYB48-1*), Os10t0409400-01 (*RD22*), Os12t0113700-00 (C3HC4 type family protein), and unknown proteins (Os02t0772100-01, Os07t0511400-01, Os12t0113600-01) (Table S4). Overexpression of *OsMYB48-1* enhanced drought and salinity tolerance by regulating stress-induced ABA synthesis in rice [32]. The tolerance to salinity and drought stresses of transgenic rice overexpressing *OsMYB48-1* was significantly improved. Overexpression of *OsMYB48-1* regulated the expression of some stress responsible genes, such as *RAB16C*, *RAB16D*, *RAB21* and *LEA3* under drought stress conditions [32]. The expression of *OsMYB48-1* was up regulated under both PEG treatment and salt treatment in transgenic rice lines compared with WT lines in this study. RD22 has also been reported to be involved in *Arabidopsis* drought tolerance and ABA-mediated stress responses in grape [33,34]. These results suggested that ABA-mediated stress responses might be activated for stress tolerance of transgenic plants.

### 3.3. The Abiotic Tolerance of Transgenic Plants Is Gained from Regulations of Several Important Components of Plant Stress Defense Networks

Abiotic stresses, such as drought and high salinity, profoundly affect growth, development, and productivity of rice, and plants develop different mechanisms to resist salt and drought stresses [35,36,37,38]. Protein kinase serves as the common mediators to regulate plant responses to multiple stresses including drought and salt stimulus. CIPK is a plant-specific family of serine-threonine kinases; activated CIPKs can subsequently transduce calcium signals by phosphorylating downstream signaling components [39]. *AtCIPK14* is induced by salt and ABA treatments, and regulates the expression of *DREB1A/DREB2A*, *RD29A/RD29B*, *RAB18* and *RD22*. T-DNA lines in which *AtCIPK14* was knocked out were sensitive to salt and ABA treatments [40]. Transgenic tobacco overexpressing *TaCIPK14*, the sequence of which is significantly similar to *OsCIPK14*, exhibited significant salinity tolerance because of higher catalase activity, as well as decreased H_2_O_2_ content and decreased Na^+^ content under salt stress [41]. The *OsCIPK14* (Os12t0113500-01) gene was up-regulated in OXR lines under both PEG and salt treatments, which could contribute to the enhanced stress tolerance of OXR lines. Transgenic rice plants overexpressing *OsCIPK5* were significantly tolerant to salt stress [42]. In our study, *OsCIPK15* (Os11t0113700-01) was up-regulated at 0 h, 24 h of PEG treatment and 0 h, 1 h of salt treatment (Tables S2 and S3). These data indicated that the difference of stress response occurred at early stage of stress signal transduction between WT and OXR lines.

TFs and corresponding *cis*-acting elements function as molecular switches in the conversion of stress signal perception to stress-responsive gene expression and are the last step of stress signal transduction [43]. DREB transcriptional factors can specially bind to DRE/CRT cis-element and activate stress inducible genes. *OsDREB1F* gene was induced by ABA application, cold, drought and salt stress. Overexpression of *OsDREB1F* conferred *Arabidopsis* and rice tolerance to low temperature, salt and drought. *OsDREB1F* not only activated *COR* (cold-regulated) genes with DRE/CRT element, but also activated *RAB18* and *RD29B* genes, which suggested *OsDREB1F* may also participate in ABA-dependent pathway [44]. In our study, the expression of *OsDREB1F* (Os04t0549700-01) was up-regulated at 6 h of PEG treatment, and 1 and 6 h of salt treatment in OXR lines when compared to WT lines.

ABA responsive element (ABRE) is one of the important cis-elements of ABA-inducible genes [45]. ABRE-binding factors (ABFs) or ABRE-binding proteins (AREBs) contain basic leucine zipper (b-zip) structure [46]. In OXR lines, the expression of *OsbZIP73* (Os09t0474000-01) gene was up-regulated at 0 h of PEG treatment and salt treatment. NAC134 TF was up-regulated at 0 h of salt treatment, 0 h and 6 h of PEG treatment in OXR lines.

All our results suggested that the stress tolerance of OXR lines was rendered by many components of the stress-regulation network, from signal transduction to stress-responsive gene expression and ROS scavenging (Table 3 and Table 4).

### 3.4. The Ethylene and Jasmonate Pathways Contribute to the Improvement of Abiotic Tolerance in Transgenic Plants

Ethylene plays significant roles in the whole life cycle of plants, ranging from growth and development to stress responses [47]. Rice has a different ethylene response phenotype in comparison with *Arabidopsis* and the other monocotyledonous species tested [48]. Meanwhile, unlike the positive roles of EIN2 and EIN3 in salinity response in *Arabidopsis*, the main positive ethylene signaling components MHZ7/OsEIN2, MHZ6/OsEIL1, and OsEIL2 all negatively regulate the salinity-tolerance of rice seedlings [48,49,50,51,52,53]. In this study, one of components of EBF1/2, *EBF1* (Os06t0605900-01) was up-regulated under both drought and salt treatments compared with WT. CTR1 was up-regulated in transgenic plants compared with WT under drought treatment (Figure 4). This may imply that repressed ethylene signal pathway enhances the drought and salinity tolerance of rice, which is consistent with previous reports for inhibition of ethylene signal transduction pathway to improve abiotic tolerance [53].

On the contrast, the JA pathway positively regulates drought tolerance and salt tolerance in plants [54,55,56,57,58]. In the JA linear signal pathway, COI1 is a positive regulator and JAZ is a negative regulator. In our study, the transcriptional level of *COI1* gene was up-regulated and *JAZ* genes were down-regulated in OXR plants as compared to WT rice plants under stress conditions (Figure 4). That means the JA signal pathway was up-regulated by ectopic expression of *AmRosea1*, which was beneficial to confer drought and salt tolerance in transgenic rice.

In the present work, AmROSEA1 overexpression in rice rendered transgenic rice salinity and drought tolerance by direct or indirect regulation of diverse sets of genes, which provides a possible way to utilize this gene to alter the abiotic tolerance in more rice cultivars.

## 4. Materials and Methods

### 4.1. Plant Material and Growth Conditions

The rice cultivar *Nipponbare* (*Oryza sativa* L. ssp. *japonica*) was used in this study. The wild type (WT) and transgenic rice plants were grown on half-strength Murashige and Skoog (MS) medium under 14 h light/12 h dark conditions at 28 °C in a greenhouse for four weeks and then transplanted into a rice paddy field. WT and the transgenic progeny plants were grown side by side in a rice paddy field at Shandong Academy of Agricultural Science, and the seeds were harvested from individual lines in 2011.

### 4.2. Plasmid Construction

Total RNA was extracted from flower buds of *A. majus* using Trizol reagent (Invitrogen, Carlsbad, CA, USA). RNA samples were treated with DNAase I. First-strand cDNA synthesis was performed with the AMV reverse transcription system kit (TaKaRa, Dalian, China) using oligo (dT)_18_ primers according to the manufacturer’s instructions. The full-length *AmRosea1* cDNA was obtained by high fidelity PCR with PrimeSTAR HS DNA polymerase (TaKaRa, Dalian, China) with a pair of primers (AW22100: ACTCGAGATGGAAAAGAATTGTCGT and AW22101: TGGATCCTTAATTTCCAATTTGTTG). A 673-bp *Xho*I/*Bam*HI*AmRosea1* PCR product was cloned into the pBluescriptII KS vector. After verification by sequencing and enzyme digestion, the 673-bp fragment was cloned into the *Xho*I and *Bam*HI site of the intermediate vector pRT101 resulting in the plasmid pRT101-*AmRosea1*. Then, the region containing CaMV 35S promoter-*AmRosea1*cDNA-CaMV terminator from plasmid pRT101-*AmRosea1* was inserted in plasmid pCAMBIA1300 at the *Pst*I site resulting in the binary construct pCAMBIA1300-*AmRosea1*.

### 4.3. Transformation of AmRosea1 and Screening Homozygote Lines

The plasmid pCAMBIA1300-*AmRosea1* was transformed into rice cultivar *Nipponbare* (*O*ryza *sativa* L. ssp. *japonica*) by the *Agrobacterium*-mediated co-cultivation method [59,60] and regenerated on MS medium containing 50 mg·L^−1^ hygromycin B. The seeds of different transgenic plants were harvested separately. To screen homozygote transgenic lines, 30 seeds of each of the T1 progenies were grown on half-strength MS medium containing 50 mg·L^−1^ hygromycin B. After incubation for seven days, the number of survived seedlings was counted.

### 4.4. PCR Analysis

Genomic DNA was extracted from leaves of the rice plant using the CTAB method [61]. The *AmRosea1* gene-specific primers, AW22100 and AW22101, were used to detect the integration of transgenic plants. PCR conformation of transgenic plants was also performed using *hygromycin phosphotransferase* (*hyt*) gene-specific primers OL561 (TTCTACACAGCCATCGGTCC) and OL562 (CCCATGTGTATCACTGGCAA). For semi-quantitative RT-PCR, total RNA was extracted from T0 transgenic lines using Trizol reagent (Invitrogen, Carlsbad, CA, USA). Contaminating DNA was removed using DNase I and 1 µg total RNA from each sample was synthesized to first-strand cDNA using the RT-PCR kit (TaKaRa, Dalian, China). The *actin* gene of rice was employed as internal control; its primers were OL591 (CCTCTCTCTGTATGCCAGTGGTCGT) and OL5925 (ATGTAGTCTCATGGATACCCGCAGC).

### 4.5. Anthocyanin and Total Flavonoid Content Quantification

Relative anthocyanin levels were determined according to the method of Neff and Chory [62], with some modifications. The whole leaves of rice at the trefoil stage and the middle section of the second and third leaves from bottom to top of the rice plant at the heading stage were taken. The midribs were removed and the leaves were ground into power in liquid nitrogen. Seeds were shattered to power with grinder (FSJ-A03D1, Guangdong Bear Electric Co., Shunde, China). To measure the anthocyanin content, 0.5 g of young leaf powder, 0.1 g of mature leaf powder, or 0.3 g of seed powder was homogenized five times for 20 s with 6 mL of methanol (1% HCl); 1 min separated each mixing period. Samples were vortexed vigorously and subsequently cleared by centrifugation for 10 min. Then, 300 μL supernatant was diluted with 200 μL of MilliQ^®^ (Millipore, Boston, MA, USA) water and 500 μL of chloroform. The mixture was vortexed vigorously for 1 min and centrifuged for 2 min. The upper aqueous phase was then aliquoted, diluted with an equal volume of methanol (1% HCl), and measured using UV spectrophotometer (Ubest, Japan Spectroscopic Co., Tokyo, Japan) at 528 nm. To measure the flavonoid content, the previously ground 0.1 g of young leaf, 0.04 g of mature leaf, or 1.0 g of ground seed samples were extracted with 20 mL of acidified methanol (methanol:water:chlorhydric acid = 79:20:1, *v*/*v*/*v*) for 30 min, agitated every five minutes. Extracts were centrifuged at 3000 rpm for 10 min, and the absorbance value of the supernatant at 305 nm was measured with a spectrophotometer [63].

### 4.6. Stress Tolerance Experiments

T2 transgenic homozygous lines were used for the stress tolerance treatment. On the seventh day after germination on half-strength MS medium, 48 seedlings of each line were transplanted into small pots containing nutritious soil (vermiculite:soil = 2:1). Four seedlings were grown per pot. One half of the seedlings were exposed to salinity stress, and the other half were exposed to drought stress. The small pots with holes on the bottom were placed in large plastic trays that held a depth of 7–8 cm of water.

For drought stress, after supplying normal water for 10 days, water was withheld from seedlings for 18 days. Stress treatment was followed by seven days of recovery by supplying tap water, and then survival rates were recorded by counting the number of live seedlings. Survival rates were determined as the number of surviving seedlings to the number of total treated seedlings.

For salt stress, after supplying tap water for 10 days, salinity stress was carried out starting from a 100-mM salt concentration at 24 hourly increments of 50 mM, and the seedlings were treated with the maximum salt concentration of 250 mM for seven days. Stress treatment was followed by 10 days of recovery by supplying the plants with normal water, and then survival rates were recorded. Survival rates were determined as the number of surviving seedlings to the number of total seedlings used.

### 4.7. cDNA Library Construction and Illumina Sequencing

Seeds were washed with distilled water and germinated in the dark at 28 °C for 3 days. Germinated seeds were transferred to 96-well plates with no bottom, floating on 1/2 MS solution in a growth chamber (16/8 h light/dark photoperiod at 25 °C with 70% RH). Seedlings at the three-leaf stage were treated with 200 mM NaCl or 15% PEG4000 for salinity and drought stress, respectively.

Twelve separate libraries were prepared from various treated plant materials. The libraries for WT plants were WT_S0, the WT plants sampled just before salt treatment; WT_S1, the WT plants at 1 h of salt treatment; WT_S6, the WT plants at 6 h of salt treatment; WT_P0, the WT plants sampled just before PEG treatment; WT_P6, the WT plants at 6 h of PEG treatment; WT_P24, the WT plants at 24 h of PEG treatment, and the responsible libraries for transgenic plants were OX_S0, OX_S1, OX_S6, OX_P0, OX_P6, OX_P24. For each treatment, the leaves of 15 plants were sampled and pooled to minimize the effect of transcriptomic unevenness among plants. Samples were frozen in liquid nitrogen and ground into fine powders. Total RNA was extracted using Trizol reagent (Invitrogen, Carlsbad, CA, USA) according to manufacturer’s instructions.

The integrity of RNA was determined by a 2100 Bioanalyzer (Agilent Technologies, Santa Clara, CA, USA), and the concentration was detected by a Nanodrop ND-1000 spectrophotometer (Thermo Fisher Scientific, Waltham, MA, USA). Qualified total RNA were treated with DNase I (TaKaRa, Dalian, China) to remove contaminant genomic DNA, and mRNA were purified from the genomic DNA removed total RNA by Dynabeads^®^ Oligo (dT)25 (Invitrogen, Carlsbad, CA, USA). To construct the sequencing library, 100 ng of purified mRNA from each sample was used as input for NEBNext^®^ UltraTM RNA Library Prep Kit for Illumina (NEB, Ipswich, MA, USA). The quality of libraries was monitored by a Nanodrop ND-1000 spectrophotometer (Thermo Fisher Scientific, Waltham, MA, USA), 2% agarose gel electrophoresis and a high-sensitivity DNA chip. Hanyu Bio-Tec Corporation (Shanghai, China) subjected 10 ng qualified libraries of each sample to cluster generation on cBot (Illumina, Santiago, CA, USA) using a TruSeq PE Cluster kit (Illumina, Santiago, CA, USA) and sequencing on aIllumina HiseqTM2500 platform (Illumina, Santiago, CA, USA).

### 4.8. Analysis of RNA-Seq Data and Identification of DEGs

Raw reads pre-processing and clean reads mapping were performed using the CLC Genomics Workbench v6.5 software (QIAGEN, Redwood, CA, USA). After quality and adapter trimming, the clean reads were mapped to the rice reference genome using the gene model annotation IRGSP-1.0.20 from RAPDB (http://rapdb.dna.affrc.go.jp/). The RPKM value of each transcript was calculated and saved for subsequent analysis. The DEGs were detected using R package DEGseq (1.24.0) by comparing the gene expression levels between two conditions. In this study, we first screened DEGs between different points in time within transgenic plants or within WT plants with fold changes ≥ 2, *p*-value < 0.05, and corrected *p*-value of false discovery rate (FDR) < 0.05; then we screened DEGs between transgenic plants and WT plants at the same time point with fold changes ≥ 2, *p*-value < 0.05, and corrected *p*-value of FDR < 0.05.

### 4.9. GO Enrichment and KEGG Mapping

GO enrichment analysis was performed using AgriGO, a web-based GO analysis toolkit (http://bioinfo.cau.edu.cn/agriGO/) [64]. Before being submitted to AgriGO, the gene IDs of DEGs were converted to MSU7.0 format by a local Python script. The target species was set as *O. sativa* MSU7.0, and Fisher’s exact test (*p* < 0.05) and multi-test adjustment (FDR < 0.05) were applied. To assignment DEGs to known metabolic or regulatory pathways, KEGG Mapper (http://www.genome.jp/kegg/mapper.html) was used, and the KEGG database for RAPDB was set as the target [65].

### 4.10. qPCR Validation of Differential Transcription

Total RNA was isolated from the leaves and stems of plants subjected to the various treatments described above. Contaminating DNA was removed using DNase I and total RNA (1 µg) from each sample was used to synthesized the single-strand cDNA using the RT-PCR kit (Tiangen, Beijing, China). qPCRs were performed in an Agilent Stratagene Mx3005p Real Time PCR system using a SuperReal PreMix Plus SYBR Green (Tiangen, Beijing, China), according to the manufacturer’s protocol. Gene-specific primers were designed using Primer5 software. Each 20 μL qPCR contained 10 μL 2 × SuperReal PreMix Plus, 0.6 μL 10 μmol sence primer and antisence primer, 4 μL diluted cDNA, 0.4 μL 50 × ROX reference dye, and 4.4 μL RNase-free double distilled water, and was exposed to an initial denaturation (95 °C/15 min), followed by 40 cycles of 95 °C/10 s and 60 °C/25 s. After amplification, all results were screened to verify a single peak melting curve for the specificity of the amplifications. Three biological replicates were performed for each sample. Relative transcript abundance was obtained by including the *Osactin1* gene as the reference, and was based on the $2^{-\Delta \Delta C_{t}}$ method [66]. Primers used for qRT-PCR are listed in Table S11.

## Figures and Tables

**Table 1:** List of DEGs related to osmotic adjustment under PEG treatment.

Gene ID	log_2_ (RPKM_OXR_/RPKM_WT_)			Description
	0 h	6 h	24 h	
Os02t0575500-01	−0.16	−1.13 *	−0.23	similar to ABC (ATP-binding cassette) transporter-like
Os09t0448200-02	1.43 **	2.62 **	1.75 **	similar to high-affinity potassium transporter
Os02t0106100-01	−1.65 *	−2.73 *	−1.61 *	similar to fructosyltransferase

* Represents down regulation; ** Represents up regulation.

**Table 2:** List of DEGs related to osmotic adjustment under salt treatment.

Gene ID	Log_2_ (RPKM_OXR_/RPKM_WT_)			Description
	0 h	1 h	6 h	
Os07t0669700-01	−0.09	1.01 **	1.09 **	potassium transporter 4
Os02t0106100-01	−1.91 *	−1.08 *	−0.84	similar to fructosyltransferase
Os03t0316200-01	−0.31	−2.83 *	0.05	similar to galactinol synthase
Os01t0170000-01	−0.73	−2.78 *	0.18	raffinose synthase family protein
Os09t0376800-01	0.38	0.77	1.33 **	similar to trehalose-6-phosphate synthase (OsTPS11)
Os02t0611200-03	−1.27 *	−1.42 *	−2.11 *	similar to s-adenosylmethionine decarboxylase 2

* Represents down regulation; ** Represents up regulation.

**Table 3:** List of DEGs related to redox regulation under PEG treatment.

Gene ID	Log_2_ (RPKM_OXR_/RPKM_WT_)			Description
	0 h	6 h	24 h	
Catalase (CAT)				
Os06t0727200-01	0.79	1.36 **	0.79	catalase isozyme B (EC 1.11.1.6)
Ascorbate peroxidase (APX)				
Os04t0602100-01	−1.77 *	−2.21 *	−1.34 *	l-ascorbate peroxidase
Peroxiredoxin (PrxR)				
Os06t0196300-01	−1.64 *	−0.49	0.77	similar to peroxiredoxin Q
Glutathione S-transferase (GST)				
Os04t0435500-01	−0.72	−1.03 *	−0.15	glutathione S-transferase, c-terminal-like domain containing protein
Os10t0530500-01	−1.42 *	−1.78 *	−2.0 *	similar to glutathione-S-transferase
Peroxidase superfamily protein				
Os07t0677200-01	−0.04	2.81 **	0.64	peroxidase
Os07t0677400-00	−1.47 *	−0.28	−0.02	peroxidase.
Os04t0688100-01	0.45	2.21 **	0.18	peroxidase (EC 1.11.1.7)
Os04t0688100-02	0.79	1.87 **	0.58	peroxidase (EC 1.11.1.7)
Os04t0688500-01	0.9	2.72 **	1.21 **	peroxidase (EC 1.11.1.7)
Os10t0109300-01	0.35	1.94 **	0.17	similar to peroxidase (EC 1.11.1.7)
Os01t0263300-01	0.44	1.42 **	−0.6	similar to peroxidase 72 precursor (EC 1.11.1.7)
Os01t0962700-01	0.47	1.11 **	−0.16	similar to peroxidase 12 precursor (EC 1.11.1.7)
Os01t0963000-01	1.01 **	4.55 **	1.38 **	similar to peroxidase BP 1 precursor
Os06t0547400-01	0.73	1.76 **	1.03 **	similar to peroxidase P7 (EC 1.11.1.7)
Os06t0695500-01	0.01	2.19 **	−0.44	similar to peroxidase 16 precursor (EC 1.11.1.7)
Os04t0688300-01	1.02 **	3.24 **	1.77 **	Haem peroxidase, plant/fungal/bacterial family protein.
Os01t0326300-01	−0.07	1.92**	0.24	Haem peroxidase, plant/fungal/bacterial family protein.
Os06t0521500-01	1.26 **	3.09 **	0.82	Haem peroxidase family protein.
Os06t0306300-01	0.42	1.94 **	0	Haem peroxidase domain containing protein.
Os02t0684400-01	0.24	1.33 **	0	similar to OXS3 (OXIDATIVE STRESS 3)
Dehydrogenase/reductase				
Os04t0531900-01	0.62	1.05 **	−0.07	short-chain dehydrogenase/reductase SDR domain-containing protein
Os11t0484500-01	0.31	−0.76	−1.23 *	similar to 6-phosphogluconate dehydrogenase

* Represents down regulation; ** Represents up regulation.

**Table 4:** List of DEGs related to redox regulation under salt treatment.

Gene ID	log_2_ (RPKM_OXR_/RPKM_WT_)			Description
	0 h	1 h	6 h	
Catalase (CAT)				
Os02t0115700-01	−0.09	1.6 **	0.67	catalase isozyme A (EC 1.11.1.6)
Os06t0727200-01	−0.46	0.76	1.04 **	catalase isozyme B (EC 1.11.1.6)
Os06t0727200-02	1.64 **	0.57	−0.31	catalase isozyme B (EC 1.11.1.6)
Ascorbate peroxidase (APX)				
Os04t0602100-01	−2.23 *	−2.37 *	−1.6 *	l-ascorbate peroxidase
Os06t0567900-01	0.67	1.15 **	−0.01	similar to ascorbate oxidase (Fragment)
Peroxiredoxin (PrxR)				
Os06t0196300-01	−1.01 *	−0.62	−0.64	similar to peroxiredoxin Q (Fragment)
Os06t0196300-02	0.04	−1.02 *	−0.07	similar to peroxiredoxin Q (Fragment)
Glutathione S-transferase (GST)				
Os01t0369700-01	−0.77	0.61	1.26 **	similar to glutathione S-transferase GST 8
Os04t0435500-01	−0.38	−1.88 *	−0.26	glutathione S-transferase, C-terminal-like domain containing protein
Peroxidase (POD)				
Os04t0688100-02	0.77	0.83	2.95 **	peroxidase (EC 1.11.1.7)
Os04t0688100-01	−0.1	1.36 **	0.18	peroxidase (EC 1.11.1.7)
Os04t0688500-01	0.44	−0.09	1.07 **	peroxidase (EC 1.11.1.7)
Os07t0677400-00	−1.08 *	−3.03 *	−0.37	peroxidase
Os03t0121200-01	−0.25	−2.39 *	−0.67	similar to Peroxidase 1

* Represents down regulation; ** Represents up regulation.

## Supplementary Materials

Supplementary materials can be found at www.mdpi.com/1422-0067/18/1/2/s1.
