# Supplementary material for: Overexpression of AmRosea1 Gene Confers Drought and Salt Tolerance in Rice
Source: Int J Mol Sci. 2016 Dec 22;18(1):2. doi: 10.3390/ijms18010002 (PMC5297637; doi:10.3390/ijms18010002)
Supplement: Supplementary file 1 [file ijms-18-00002-s001.zip › ijms-158741-Supplementary Materials/ijms-158741-Table S1, S5-S11, Figure S1-S2-done.pdf]

Supplementary Materials: Overexpression of *AmRosea1* Gene Confers Drought and Salt Tolerance in Rice

Mingzhu Dou, Sanhong Fan, Suxin Yang, Rongfeng Huang, Huiyun Yu and Xianzhong Feng

Table S1. Summary of mapping results.

**Map to Gene**	**WT-PEG-0 h**		**WT-PEG-6 h**		**WT-PEG-24 h**		**WT-Salt-0 h**		**WT-Salt-1 h**		**WT-Salt-6 h**	
	**Reads Number**	**Percentage**	**Reads Number**	**Percentage**	**Reads Number**	**Percentage**	**Reads Number**	**Percentage**	**Reads Number**	**Percentage**	**Reads Number**	**Percentage**
clean reads	7,333,513	100%	7,116,841	100%	7,389,213	100%	7,387,204	100%	7,239,102	100%	7,390,469	100%
Total mapped reads	6,596,369	89.95%	6,432,701	90.39%	6,265,857	84.80%	66,69,882	90.29%	6,607,019	91.27%	6,768,117	91.58%
Total unmapped reads	737,144	10.05%	684,140	9.61%	1,123,356	15.20%	717,322	9.71%	632,083	8.73%	622,352	8.42%
Unique_match	3,293,165	44.91%	3,283,291	46.13%	3,168,963	42.89%	3,314,077	44.86%	3,409,482	47.10%	3,656,368	49.47%
Multi-position match	3,303,204	45.04%	3,149,410	44.25%	3,096,894	41.91%	3,355,805	45.43%	3,197,537	44.17%	3,111,749	42.10%
Perfect match	6,040,500	82.37%	5,874,905	82.55%	5,711,530	77.30%	6,129,419	82.97%	6,048,954	83.56%	6,180,542	83.63%
≤5 bp mismatch	525,415	7.16%	530,256	7.45%	522,317	7.07%	505,249	6.84%	526,733	7.28%	548,742	7.42%
**Map to Gene**	**OXR-PEG-0h**		**OXR-PEG-6h**		**OXR-PEG-24h**		**OXR-Salt-0h**		**OXR-Salt-1 h**		**OXR-Salt-6 h**	
	**Reads Number**	**Percentage**	**Reads Number**	**Percentage**	**Reads Number**	**Percentage**	**Reads Number**	**Percentage**	**Reads Number**	**Percentage**	**Reads Number**	**Percentage**
clean reads	7,245,902	100%	7,321,939	100%	7,401,080	100%	7,221,670	100%	7,336,233	100%	7,344,809	100%
Total mapped reads	6,449,341	89.01%	6,556,674	89.55%	6,598,040	89.15%	6,501,240	90.02%	6,173,666	84.15%	6,639,746	90.40%
Total unmapped reads	796,561	10.99%	765,265	10.45%	803,040	10.85%	720,430	9.98%	1,162,567	15.85%	705,063	9.60%
Unique_match	3,239,357	44.71%	3,309,357	45.20%	3,343,701	45.18%	3,246,232	44.95%	3,208,032	43.73%	3,554,091	48.39%
Multi-position match	3,209,984	44.30%	3,247,317	44.35%	3,254,339	43.97%	3,255,008	45.07%	2,965,634	40.42%	3,085,655	42.01%
Perfect match	5,885,693	81.23%	6,009,162	82.07%	6,075,612	82.09%	5,954,529	82.45%	5,635,346	76.82%	6,034,068	82.15%
≤5 bp mismatch	529,401	7.31%	512,213	7.00%	484,554	6.55%	514,871	7.13%	502,769	6.85%	559,694	7.62%

Table S5. List of DEGs involved in plant hormone pathways under drought stress.

Hormone	EC	Gene ID	log_2 ($RPKM_{OXR}/RPKM_{WT}$)		
			0 h	6 h	24 h
Cytokinine	A-ARR	Os04t0673300-01	1.238085	3.065659	0.979313
Abscisic acid	PYR/PYL	Os03t0297600-01	0.563933	1.987756	−0.19252
		Os05t0473000-01	1.387322	4.935228	0.705962
	PP2C	Os01t0583100-01	−0.170357	−1.130925	0.161511
	ABF	Os02t0766700-01	−0.064178	−1.153477	0.430554
Ethylene	EBF1/2	Os06t0605900-01	1.120422	2.458976	0.638164
Jasmonic acid	COI1	Os05t0449500-01	0.676641	1.037637	0.484258
Salicylic acid	PR-1	Os07t0129300-00	2.452397	0.953668	1.374756

Table S6. List of DEGs involved in plant hormone pathways under salt stress.

Hormone	EC	Gene_ID	log_2 ($RPKM_{OXR}/RPKM_{WT}$)		
			0 h	1 h	6 h
Cytokinine	A-ARR	Os04t0673300-01	0.764954	−1.070037	−0.241291
		Os11t0143300-01	−2.214695	−1.208195	2.157301
		Os12t0139400-01	−2.050575	−1.877847	0.362545
Abscisic acid	SnRK2	Os07t0622000-01	−0.437177	−1.439286	0.192399
Ethylene	CTR1	Os02t0527600-01	0.353983	1.167067	0.133855
	EBF1/2	Os06t0605900-01	1.068686	0.48677	0.007147
Jasmonic acid	JAZ	Os03t0402800-01	−0.878805	−1.461285	−0.143723
		Os07t0615200-01	−0.687653	−1.525973	−0.237743
Salicylic acid	PR-1	Os07t0129300-00	−1.130328	−0.249022	−1.460031

Table S7. List of DEGs related stress tolerance in OXR plants compared with WT under drought condition.

Gene ID	log_2 ($RPKM_{OXR}/RPKM_{WT}$)			Description
	0 h	6 h	24 h	
HSP (heat shock protein)				
Os01t0135900-02	−0.26	1.15	0.36	LMW heat shock protein.
Os01t0184100-01	1.08	2.14	−0.46	Similar to 17.5 kDa class II heat shock protein.
Os06t0650900-02	0.99	−1.25	0.17	Heat shock protein DnaJ family protein.
Os06t0716700-01	−2.92	2.69	0.68	Similar to Heat shock protein 90.
Os07t0517100-03	0.20	1.49	−0.27	Similar to 18.8 kDa class V heat shock protein.
Os11t0216100-01	−0.09	−1.00	−0.89	Similar to Heat shock protein binding protein.
LEA (late embryogenesis abundant)				
Os01t0314800-01	−0.61	−1.21	0.62	Late embryogenesis abundant protein 3 family protein.
Os05t0542500-02	0.21	3.36	2.41	LEA-like protein.
Chaperone protein				
Os08t0452900-01	0.09	1.26	0.12	Similar to Chaperone protein dnaJ.
Os12t0277500-01	−0.65	0.39	1.00	60 kDa chaperonin alpha subunit.
Metallothionein				
Os01t0974200-01	−0.49	−1.19	−1.13	RicMT (Metallothionein-like protein).
Os05t0202800-00	0.39	−4.34	NA	Similar to Metallothionein-like protein 3B.

Table S8. List of DEGs related stress tolerance in OXR plants compared with WT under salt condition.

Gene ID	log_2 ($RPKM_{OXR}/RPKM_{WT}$)			Description
	0 h	1 h	6 h	
HSP (heat shock protein)				
Os01t0135900-02	−0.36	0.32	1.14	LMW heat shock protein.
Os03t0266300-01	−0.48	1.08	−0.43	Class I low-molecular-weight heat shock protein 17.9.
Os03t0266900-02	−0.63	1.24	−0.13	Low molecular mass heat shock protein Oshsp17.3.
Os05t0579900-02	0.03	−1.36	0.42	Heat shock protein DnaJ.
Os06t0253100-01	−0.86	2.57	−0.65	Heat shock protein Hsp20 domain containing protein.
Os06t0716700-01	−0.57	3.6	2.08	Similar to Heat shock protein 90.
Os08t0500700-01	−0.71	1.03	−0.12	Similar to Heat shock protein 82.
LEA (late embryogenesis abundant)				
Os01t0314800-01	−0.99	−1.44	−0.42	Late embryogenesis abundant protein 3 family protein.
Os01t0705200-01	NA	−2.09	−0.37	Late embryogenesis abundant protein repeat containing protein.
Os03t0322900-00	−1.6	−2.14	−0.32	Late embryogenesis abundant protein repeat containing protein.
Os08t0327700-01	NA	−2.09	−0.8	Late embryogenesis abundant (LEA) group 1 family protein.
Os05t0542500-02	2.02	−0.65	−0.23	LEA-like protein.
Chaperone protein				
Os08t0405700-01	0.66	−1.12	−0.43	Similar to Copper chaperone homolog CCH.
Metallothionein				
Os12t0570700-01	0.49	1.46	0.29	Similar to Metallothionein-like protein type 1.
PIP (plasma membrane intrinsic protein)				
Os02t0629200-02	−0.46	−1.12	−0.49	Similar to HvPIP2;1 protein.

Table S9. Expressions of anthocyanin relating genes in OXR and WT plants under drought stress

Gene ID	OXR (RPKM)			WT (RPKM)		
	0 h	6 h	24 h	0 h	6 h	24 h
F3H						
OS02T0767300-01	1.406	0.667	1.527	2.659	0.602	0.771
Os03t0122300-01	142.858	188.527	245.626	210.445	156.028	297.271
Os04t0581000-01	4.058	3.429	8.436	1.735	0.648	3.459
F3′H						
Os10t0317900-01	37.962	14.645	17.149	48.349	19.766	26.152
Os10t0320100-01	8.212	0.755	1.481	25.071	0.553	1.937
FLS						
OS02T0767300-01	1.406	0.667	1.527	2.659	0.602	0.771
DFR						
OS01T0633500-00	0	0	0	0	0	0
Os03t0184550-01	15.324	19.105	21.783	3.696	8.042	19.385
OS03T0184600-01	13.05	10.801	14.496	10.672	11.117	13.673
OS08T0183900-01	13.821	11.787	3.293	23.287	27.634	9.186
Os08t0515900-01	0.701	1.16	0.701	0.636	0.461	0.614
Os09t0491868-00	0	0	0	0	0	0
UFGT						
Os06t0192100-00	0.377	0.312	0.063	0.559	0.619	0.264
Os07t0148200-01	6.002	5.865	3.533	8.74	6.007	4.794

Table S10. Expressions of anthocyanin relating genes in OXR and WT plants under salt stress.

Gene ID	OXR (RPKM)			WT (RPKM)		
	0 h	1 h	6 h	0 h	1 h	6 h
F3H						
OS02T0767300-01	1.624	0.129	0	0.962	0.362	0
Os03t0122300-01	122.342	172.764	91.472	174.902	354.6	113.196
Os04t0581000-01	3.723	3.016	0.648	3.99	6.655	0.427
F3′H						
Os10t0317900-01	19.664	7.843	2.046	37.843	7.572	0.649
Os10t0320100-01	1.971	0.847	1.421	5.476	0.791	1.988
FLS						
OS02T0767300-01	1.624	0.129	0	0.962	0.362	0
DFR						
OS01T0633500-00	0	0	0	0	0	0
Os03t0184550-01	48.98	82.765	48.436	27.077	18.01	44.685
OS03T0184600-01	15.193	15.91	11.596	13.312	12.659	14.467
OS08T0183900-01	8.599	10.503	18.592	14.881	10.973	22.537
Os08t0515900-01	0.863	0.865	1.037	0.517	0.577	0.626
Os09t0491868-00	0	0	0	0	0	0
UFGT						
Os06t0192100-00	0.247	0.266	0	0.185	0.186	0
Os07t0148200-01	3.251	2.362	5.685	3.981	6.014	6.883

Table S11. Primers for qRT-PCR.

Gene ID	Primer F (5′-3′)	Primer R (5′-3′)	Gene Name
Os10t0409400-01	CGGTGCGTCACATCAGCAG	TTTATTCCCTTGACCTTACCAATCA	RD22
Os06T0716700-01	CACAATGACGACAAACAGCATG	CAGGGGTTCATTCCATGTATCC	Heat shock protein (HSP90)
Os05T0542500-02	CGTCCTCCAACAGGCGAGT	CTCGTCTTCGGTCATCCCCA	LEA-like protein
Os11T0454300-01	GCTCCAGCTCAAGCTCGTCTG	CTGCTGCTCGCCCTTGTTG	Water-stress inducible protein RAB16A
Os06t0727200-02	GTCGGAGAGCGAGGCCCTA	AGGGATACGCTCCCTGTCAAACT	Catalase (CAT)
Os10t0419400-01	AGCCAGAGGTTACTCTTATGTGGAC	CGGTATGCAGGTGTTCTTCAAAGA	Submergence-induced protein 2 (Sip2)
Os04t0549700-01	GCAGACGCGAGCCATACACC	GAGCTCTTGGGCCGGTCTTG	Dehydration responsive element binding protein 1F (DREB1F)
Os01t0975300-01	GATCAACGCTGGTTGTTGGATC	AGCTCTTCCCGCTGCGCT	MYB48
Os12t0156100-01	CGCGAGGATTGTCAGTGATCC	GGAAGGATGGTAGCCGTAGACGT	NAC134
Os12t0113500-01	GGAATCTGGAGTCAAACCAGAGTG	GTCTGATCTGCTCCGAAAGCC	CIPK14

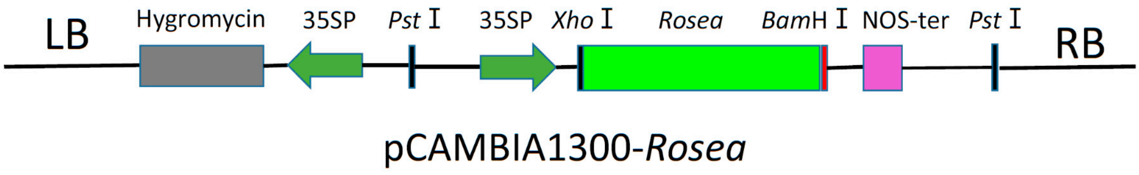

Figure S1. The sketch of the binary vector pCAMBIA1300-*AmRosea1*.

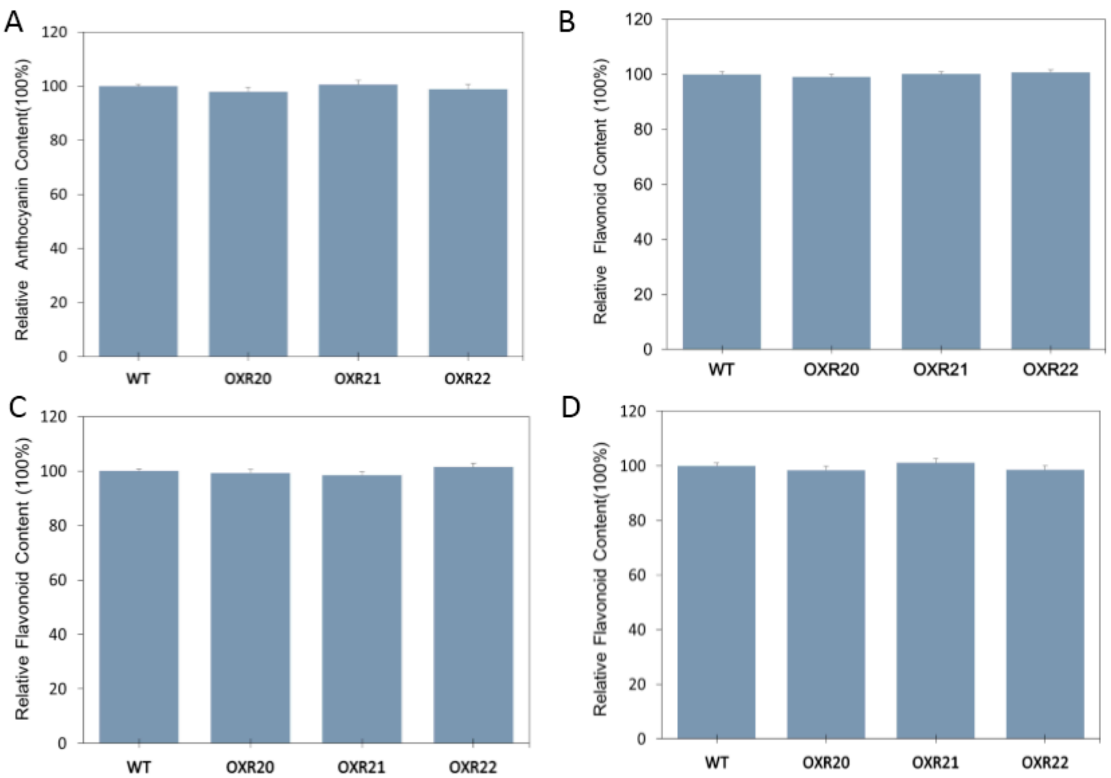

Figure S2. The relative contents of anthocyanin and flavonoids in leaves and seeds of WT and transgenic plants. (**A**) Anthocyanin of leaves; (**B**) anthocyanin of seeds; (**C**) flavonoids of leaves; (**D**) flavonoids of seeds.
